# The Role of SIRT1 in Diabetic Kidney Disease

**DOI:** 10.3389/fendo.2014.00166

**Published:** 2014-10-09

**Authors:** Rabi Yacoub, Kyung Lee, John Cijiang He

**Affiliations:** ^1^Division of Nephrology, Department of Medicine, Icahn School of Medicine at Mount Sinai, New York, NY, USA

**Keywords:** SIRT1, diabetic kidney disease, senescence, deacetylation, apoptosis, autophagy, diabetes mellitus, sirtuin

## Abstract

Sirtuins (SIRTs) are members of the silent information regulator 2 family. In mammals, of the seven known SIRTs, SIRT1 function is most studied and has been shown to regulate wide range of cellular functions that affect metabolic homeostasis and aging. SIRT1 exerts anti-apoptotic, anti-oxidative, and anti-inflammatory effects against cellular injury, and protects the cells through the regulation of mitochondrial biogenesis, autophagy, and metabolism in response to the cellular energy and redox status. SIRT1 also promotes vasodilation and protects vascular tissues. In humans and animal models with diabetic kidney disease (DKD), its expression tends to be decreased in renal cells, and increased expression of SIRT1 was found to play a renal protective role in animal models with DKD. In this review, we discuss the role and potential mechanisms by which SIRT1 protects against DKD.

## Introduction

Diabetes mellitus (DM) is a major medical problem worldwide. It is the underlying cause of microvascular disorders such as diabetic nephropathy and retinopathy and macrovascular diseases such as coronary artery and peripheral vascular diseases. Currently, more than 347 million people worldwide are suffering from DM (1), and the World Health Organization projects that it will be the seventh leading cause of death by 2030. The increased prevalence of DM has led to a significant increase in the prevalence of diabetic kidney disease (DKD) with estimates that 44% of all new end stage renal disease (ESRD) cases in US are due to DKD (2, 3). Several factors including hyperglycemia, insulin resistance, renal lipid accumulation, inflammation, and activation of the renin–angiotensin system (RAS) are involved in the pathogenesis of DKD (4) and they activate multiple signaling pathways resulting in kidney cell injury and the development and progression of the disease (5, 6).

Since the discovery of the silent information regulator 2 (Sir2) family and its beneficial effects on aging (7, 8), scientists have shown that the homologs of Sir2 in higher eukaryotic organisms, known as Sirtuins (SIRTs), are a conserved family of a nicotinamide adenine dinucleotide (NAD^+^)-dependent deacetylases/mono-ADP ribosyltransferases that are associated with numerous cellular signaling pathways that include senescence (9–12), apoptosis (13), DNA damage repair (14), and autophagy (12, 15). By far, SIRT1 is the most studied member of this family and its protective roles against kidney injury are well established, making it a promising candidate for targeted therapies to halt disease progression.

## General Cellular Functions of SIRT1

**Figure 1:**
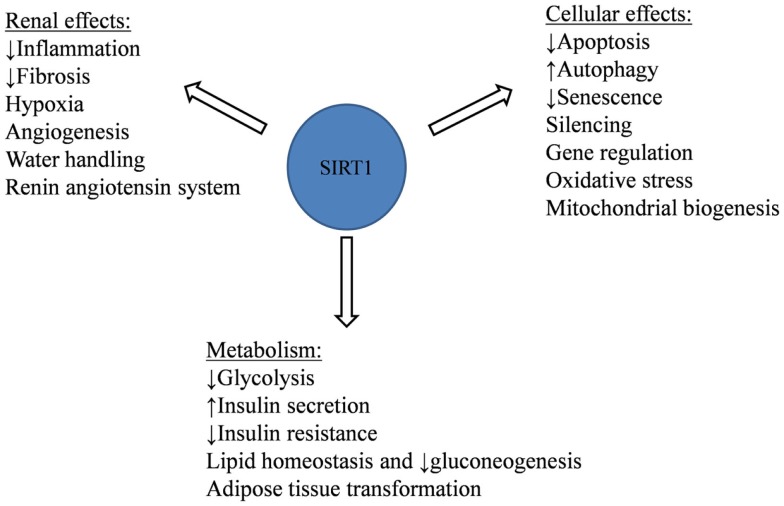
**SIRT1 cellular, renal, and metabolic protective effects against aging-related and metabolic diseases**.

SIRT1 exerts its cytoprotective effects through various mechanisms. It has anti-apoptotic, anti-oxidative, and anti-inflammatory effects, along with its regulation of mitochondrial biogenesis and autophagy (Figure 1).

### Cellular senescence, oxidative stress, and mitochondrial dysfunction

Aging is considered the most important contributor to the etiologies of metabolic decline and related diseases (16). This process is thought to be due mainly to the accumulation of oxidative stress related mitochondrial DNA (mtDNA) damages, leading to mitochondrial dysfunction (17). Increasing Sirt1 expression by calorie restriction (CR) in mice abrogated the dysmorphic mitochondrial appearances associated with aging (e.g., mitochondrial swelling and disintegration of cristae), whereas decreasing Sirt1 expression resulted in an early aging phenotype in mice, revealing the important role of SIRT1 on cellular senescence and other aging-related diseases (9–12).

Increased oxidative stress has been associated with aging, and SIRT1 has been shown to combat oxidative stress by modulating transcriptional activities of several key proteins involved in oxidative stress response and mitochondrial biogenesis. Peroxisome proliferator-activated receptor-gamma coactivator-1α (PGC-1α) is a transcriptional factor involved in lipid homeostasis and in mitochondrial biogenesis, which prevents and protects against oxidative stress (18). PGC-1α activity appears to be regulated by SIRT1 deacetylation in a tissue specific manner (19). Deacetylation of PGC-1α by SIRT1 has been observed in skeletal muscles, where PGC-1α deacetylation increases mitochondrial biogenesis, mass, and improves exercise endurance (20), and in brown adipose tissue (BAT), and endothelial cells (21).

Recent evidence suggests that SIRT1 can regulate the expression of a longevity gene p66^Shc^. p66^Shc^ is one of three isoforms encoded by the proto-oncogene SHC (Src homologous and collagen) and has been shown to promote oxidative stress, leading to mitochondrial dysfunction, senescence, and apoptosis (22, 23). Deletion of p66^Shc^ in Akita diabetic mice showed a renoprotective phenotype that included the attenuation of oxidative stress and glomerular/tubular injury and reduction in albuminuria (24, 25).

Several studies have shown that SIRT1 negatively regulates expression of *p66^Shc^* through deacetylation of histone H3 on its promoter (26). Treating different cell lines that do not usually express p66^Shc^ with histone deacetylase inhibitors induced p66^Shc^ expression (23, 26), and deletion of p66^Shc^ in Akita diabetic mice resulted in upregulation in SIRT1 expression in mice kidneys and ameliorated kidney fibrosis and preserved podocytes mass and function (24).

### Autophagy and apoptosis

Autophagy and apoptosis are two closely related processes that are triggered by common upstream signaling pathways to constitute a stress adaptation, where in general autophagy inhibits apoptosis to maintain survival (27, 28). SIRT1 exerts an anti-apoptotic and pro-autophagic responses in cells under stress conditions by directly deacetylating essential autophagy proteins (Atg), such as Atg5, Atg7, and Atg8 (29), and by deacetylation of transcription factors, such as FOXO3a, to increase the expression of autophagy proteins (12, 15). Deacetylation of FOXO3a by SIRT1 also prevents apoptosis by enhancing the expression of p27^Kip1^, a cyclin-dependent kinase inhibitor that causes G1 arrest to maintain cell viability (12, 30).

In addition, SIRT1 has been shown to deacetylate and inactivate the transcriptional activity of p53, a tumor suppressor responsible of maintaining cellular integrity by inducing cell-cycle arrest, and if necessary evoking apoptotic cell death (31). With aging, the depletion of NAD^+^ storage attenuates SIRT1 activity, leading to hyperacetylation of p53. p53 has been shown to stimulate or repress autophagy depending on its subcellular localization, where cytoplasmic p53 promotes apoptosis and inhibits autophagy (32). While it is known that SIRT1 regulates p53 function by deacetylation, whether it affects its cytoplasmic localization is not known (33).

### Adipose tissue transformation

One of the mechanism by which CR is thought to extend life span is through fat mobilization. Upon CR, SIRT1 binds and represses the fat regulator peroxisome proliferator-activated receptor-gamma (PPARγ), attenuating adipogenesis, and triggering lipolysis (34). It also selectively decreases white adipose genes *Angiotensinogen* (*Agt*), *Resistin*, *Wdnm1L*, *Chemerin*, and *Pank3* (35). PPARγ deacetylation by SIRT1 causes also a lipid transformation from white adipose tissue (WAT) to BAT through regulating ligand-dependent coactivator/corepressor exchange at the PPARγ transcriptional complex (35). WAT distribution affects metabolic risk and is linked to metabolic diseases as obesity, diabetes, and dyslipidemia (36). The metabolic benefits of this conversion include prevention of diet-induced obesity and increased insulin sensitivity (37).

## Role of SIRT1 in DM

It is well established that the risk of micro and macrovascular complications in patients with DM is closely related to the glycemic control. In the fasting state, hyperglycemia is directly related to hepatic glucose production, which in turn, along with the decreased insulin production or increased insulin resistance, is responsible for the hyperglycemia in the postprandial state (38). SIRT1 participates in regulating glucose homeostasis through regulating hepatic glucose production, lipid metabolism and insulin production, and sensitivity (39–42).

### Hyperglycemia

SIRT1 decreases hepatic glucose production via deacetylation and activation of the AMPK kinase LKB1 (39). When activated, AMPK switches off hepatic glucose, cholesterol, and triglyceride productions and promotes fatty acid oxidation. AMPK in turn also activates SIRT1 via increasing its substrate NAD^+^ (43). This reciprocal activation/dynamic interaction between AMPK and SIRT1 is disrupted by hyperglycemia, which decreases AMPK expression, leading to reduced SIRT1 expression (44).

### Lipid metabolism and insulin production and sensitivity

Under the fasting state, hepatic SIRT1 regulates lipid homeostasis and gluconeogenesis by positively regulating PPARα and its coactivator PGC-1α (10). In addition, SIRT1 also suppresses glycolysis via deacetylation of phosphoglycerate mutase-1 (PGAM1) and decreasing the expression of glycolysis genes *glucokinase* (*GK*) and *liver pyruvate kinase* (*LPK*), while PGC-1α increases the expressions of *GK* and *LPK* (10, 40). In contrast, under the feeding state hepatic SIRT1 negatively regulates gluconeogenesis via mTorc2/Akt signaling pathway, resulting in decreased transcription of gluconeogenic genes glucose-6-phosphatase (*G6pase*) and phosphoenolpyruvate carboxykinase (*Pepck*) (45). Experimental mice model of hepatic *Sirt1* deficiency displayed hyperglycemia, glucose intolerance, hepatic insulin resistance, and oxidative stress in insulin-sensitive organs through disrupted mTorc2/Akt signaling (45). This bimodal regulation of gluconeogenesis under feeding/fasting states by SIRT1 promotes adaptation to nutrient deprivation (10, 41).

SIRT1 also enhances insulin secretion from the pancreatic beta cells by regulating the expression of uncoupling protein 2 (Ucp2) (42), and decreases insulin resistance via reducing the expression of the proteins in the insulin receptor signaling pathway, such as protein tyrosine phosphatase 1B (PTP1B), and by inhibiting insulin-induced IRS-2 (insulin receptor substrate-2) tyrosine phosphorylation by deacetylation (46, 47).

## Expression of SIRT1 in Diabetic Kidney

SIRT1 expression changes under different physiological and morbid conditions. It is decreased in conditions of chronic metabolic stress, oxidative stress, or hypoxia that drives the pathophysiologies of age-related diseases including diabetes, cardiovascular, and renal diseases. In aging kidneys both the expression and activity of SIRT1 is decreased due to age-associated reduction in systemic NAD^+^ biosynthesis (12). Similarly, reduction in SIRT1 expression was observed in kidney glomeruli and tubulointerstitial compartments of patients with mild to severe DKD, which was inversely correlated with the histopathological severity of the renal disease and with the amount of proteinuria (48, 49). Experimental mouse models of DM similarly showed a loss of Sirt1 in renal proximal tubules (PT) and podocytes (48, 49), where the loss of PT Sirt1 preceded the loss of podocyte Sirt1 and the concomitant albuminuria. Interestingly, restoration of PT Sirt1 was sufficient to increase pore densities in podocytes and to mitigate albuminuria and worsening of DKD (49), suggesting that PT Sirt1 confers protection in maintaining the glomerular structure and function during the early stages of DKD and that therapeutic agents that increase the renal SIRT1 expression and activity may have a favorable impact in slowing the disease progression of DKD.

## Role and Cellular Mechanisms of SIRT1 in DKD

Among many studies indicating SIRT1’s protective role in numerous different cell types, several studies have described its protective role in different kidney cells. Some of these effects are in the general context of SIRT1’s cytoprotective actions, while others seem to have a unique specificity to specialized renal cells. In the following section, we will discuss the deleterious effects of reduced SIRT1 expression on kidney cells, and the mechanisms by which SIRT1 is found to exert its benefits on renal cells and on the concomitant inflammation, angiogenesis, and fibrosis, all of which contributes to the progression of DKD (Table 1).

**Table 1:** **Role and cellular mechanisms of SIRT1 in DKD**.

Renal cell/compartment	SIRT1 role	Mechanism	Reference
Podocytes	Anti-apoptosis	Deacetylates FOXO4, decreasing the expression of the pro-apoptotic gene Bcl2l11 (Bim)	(48)
	Decreases albuminuria	Negatively regulating Claudin-1	(49)
Proximal tubular cells	Decreases apoptosis and improves autophagy	Deacetylates FOXO3a leading to enhanced expression of Bnip3 (pro-autophagy) and p27Kip1 (anti-apoptosis)	(12)
	Attenuates hypoxia-associated mitochondrial damage	Decreases age-associated mtDNA oxidative damages	(12)
	Decreases albuminuria	Maintains the glomerular structure through suppressing Claudin-1 expression in podocytes	(49)
	Decreases fibrosis	Prevents TGF-β1 induced fibrotic response via Smad3 deacetylation	(50)
Mesangial cells	Anti-apoptosis	Attenuates TGF-β1 induced mesangial cell apoptosis through its direct interaction and deacetylation of Smad7	(51)
	Inhibition of ROS-mediated apoptosis	P53 deacetylation	(52)
	Decreases mesangial expansion	Prevents hyperglycemia-induced hypertrophy by augmenting the AMPK–mTOR signaling pathway	(53)
		Binds and activates ACE2 promoter leading to increased Ang1–7 production	(4)
Renal medulla	Protects against oxidative injury	Stabilizes HIF-1α and regulates COX2 during intermittent hypoxia-reoxygenation	(54)
	Reduces apoptosis and fibrosis	Regulates COX2 decreasing oxidative stress-induced apoptosis	(54)
Collecting ducts	Solute and water handling	Represses α-ENaC transcription	(55)
Endothelial cells	Prevents early senescence and fibrosis	Upregulates MMP-14 leading to increased matrilytic activity and angiogenesis	(56)
	Modulates angiogenesis	Prevents increased permeability and cellular junction disruption via downregulation of VEGF and Flk-1 (in podocytes too)	(57)
	Promotes vasodilatation	Decreases the expression of AT1R, and increases NO by deacetylating eNOS	(58)
Glomeruli	Attenuates hypoxia-associated mitochondrial damage	Decreases age-associated mtDNA oxidative damages	(12)
Renal cortex	Anti-inflammatory	Decreases macrophages infiltrates, deacetylates NF-κB p65 subunit and negatively regulates MCP-1, ICAM-1, and VCAM-1	(59)
Glomerular/tubular compartments	Decreases cellular senescence and apoptosis	Deacetylates H3 on p66Shc promoter	(24)

*FOXO, forkhead box protein O; Bnip3, BCL2/adenovirus E1B 19 kDa protein-interacting protein 3; ACE2, angiotensin-converting enzyme 2; Ang1–7, angiotensin-(1–7); COX2, cyclooxygenase 2; HIF-1α, hypoxia-inducible factor 1α; α-ENaC, α-epithelial sodium channel; MMP-14, matrix metalloproteinase-14;VEGF, vascular endothelial growth factor; Flk-1, fetal liver kinase-1; AT1R, angiotensin II receptor-type 1; NO, nitric oxide; eNOS, endothelial nitric oxide synthase; NF - κB, nuclear factor kappa B; MCP-1, monocyte chemotactic protein-1; ICAM-1, intercellular adhesion molecule-1; VCAM-1, vascular cell adhesion molecule-1*.

### Podocytes

One of the earliest changes in DKD is the loss of podocytes, leading to proteinuria and further kidney damage (60–62). In DKD, podocyte apoptosis is aggravated by hyperglycemia via increasing the production of advanced glycation end products (AGEs), which in turn increases FOXO4 acetylation and suppresses SIRT1 expression (48). This decreased SIRT1 expression leads to the accumulation of acetylated FOXO4 and to the expression of the pro-apoptotic gene Bcl2l11 (also known as Bim), resulting in apoptosis (48). Hyperglycemia also stimulates the generation of intracellular reactive oxygen species (ROS) through NADPH oxidase and mitochondrial pathways, leading to activation of the pro-apoptotic p38 mitogen-activated protein kinase (p38 MAPK) and Caspase 3 in podocytes (5).

### Proximal tubular cells

Tubular SIRT1 has been shown to affect podocyte function via maintaining a high glomerular nicotinamide mononucleotide (NMN) concentration via diffusion of the NMN produced in the PTs (49, 63). Decreased expression of NMN has been observed in DKD (64), which is likely to be the first pathological changes preceding proteinuria (65). PT SIRT1 communicates with podocytes through the release of NMN. PT SIRT1 appears to negatively modulate the expression of the tight junction protein Claudin-1 (49). In healthy conditions, Claudin-1 is not expressed in podocytes but primarily expressed on glomerular parietal cells. However, in diabetic mice models podocytes express Claudin-1, possibly as a part of a podocyte dedifferentiation mechanism that occurs in DKD or by reorganization of the podocyte actin cytoskeleton (63). SIRT1 also protects PTs under hypoxic conditions by inducing autophagy and inhibiting apoptosis (12, 24).

### Renal medulla

In a healthy individual, a quarter of the cardiac output is directed to the kidneys, with most of this flow going to the cortex to optimize glomerular filtration. However, the renal medulla’s blood flow is low to preserve osmotic gradient and enhance concentration ability. The renal medulla is also under chronic and constant oxidative stress due to the rapid change in interstitial tonicity and the low oxygen tension (66, 67). A small percentage of oxygen consumed by the mitochondria is incompletely reduced to ROS, which then targets the other mitochondrial components and augments the generation of increased ROS by the injured mitochondria. Under these conditions, renal mitochondria undergo a constant autophagy process. To cope with hypoxia, higher organisms’ adaptive mechanisms includes switching energy metabolism from oxygen phosphorylation to HIF-1 mediated anaerobic glycolysis (68), which concurrently blocks mitochondrial energy metabolism and biogenesis (69). SIRT1 is normally expressed in the inner medulla and is upregulated during intermittent hypoxia-reoxygenation and protects against oxidative stress via stabilizing HIF-1α and regulating of cyclooxygenase 2 (COX2) (54, 70). However in chronic hypoxic state, the renal medulla endures in DKD, SIRT1 activity is inhibited due to decreased NAD^+^ (58).

### Mesangial cells

Mesangial injury and expansion mark early histological changes in DKD, and they are correlated closely with the degree of albuminuria. SIRT1 attenuates TGF-β1 induced mesangial cell apoptosis through its direct interaction and deacetylation of Smad7, enhancing its ubiquitin-mediated proteasome degradation (51). SIRT1 also prevents high glucose-induced mesangial cells hypertrophy by augmenting the AMPK–mTOR signaling pathway (53) and subsequently blocking the activation of Akt signaling (71). NAD^+^ treatment mitigates the high glucose-induced Akt and mTOR phosphorylation in cultured mesangial cells (53).

### Endothelial cells and angiogenesis

As in diabetic retinopathy, new vessel formation is observed in DKD patients (72) and in animal models (73), contributing to its pathogenesis. Early on in DKD, there is an increase in endothelial cells number, caused by the imbalance between proliferation and apoptosis, where VEGF-A appears to be the major driver of this imbalance. Other factors affecting angiogenesis include nitric oxide deficiency, glomerular hypertension (74), altered expressions of VEGF receptors 1 and 2, Angiopoietin 2, and Tie-2 (57, 75, 76). *In vivo* and *in vitro* studies have shown that resveratrol (RSV), a SIRT1 activator, downregulates high glucose-induced VEGF-A and Flk-1 (VEGFR-2) expressions in both glomerular podocytes and endothelial cells. RSV also inhibits VEGF-A induced increased permeability and cellular junction disruption of cultured endothelial cells (57). SIRT1 also maintains endothelial cells function and prevents early senescence via upregulating matrix metalloproteinase-14 (MMP-14), an important factor for endothelial cells regeneration after injury. MMP-14 cleavage products serve as a ligand for epidermal growth factor (EGF) receptors (56). Endothelial cells have also been shown to express early senescence features in presence of high glucose through the down regulation of SIRT1 expression, leading to an increased acetylation of FOXO1 by p300 (77).

### Inflammation

Microinflammation of the glomeruli and tubulointerstitial regions and subsequent extracellular matrix expansion are common pathways for the progression of DKD, which occurs in response to renal damage as a defense mechanism. This dynamic process of pro-inflammatory macrophages M1 and anti-inflammatory macrophages M2 recruitment to the kidneys eventually leads to kidney fibrosis, highlighting the importance of inflammation as a therapy target to slow the DKD progression (78). SIRT1 can deacetylate NF-κB p65 subunit and negatively regulate the NF-κB signaling mediated expression of the inflammation-related proteins monocyte chemotactic protein-1 (MCP-1), intercellular adhesion molecule-1 (ICAM-1), and vascular cell adhesion protein-1 (VCAM-1) (59). Dietary restriction in Wistar fatty rates restores Sirt1 expression and ameliorates diabetic nephropathy abnormalities (i.e., albuminuria, mesangial matrix expansion, and renal fibrosis), which is in part mediated by Sirt1’s anti-inflammatory effects as evidenced by decreased macrophages infiltrates and changes in expression of NF-κB p65, MCP-1, ICAM-1, and VCAM-1 (59).

### Fibrosis

Kidney fibrosis is the final outcome of progressive DKD, and it results in a significant destruction of normal kidney structure accompanied by functional deterioration. TGF-β1 is upregulated in response to various kidney injury stimuli, causing renal fibrosis and epithelial–mesenchymal transformation of the renal tubules (79). Recent studies have identified Smad2 and Smad3 acetylation in response to TGF-β1 stimulation (50, 80, 81) and shown that the RSV treatment of cultured PTs leads to deacetylation of Smad3 (50). In addition, RSV administration abolished TGF-β1/Smad3 induced up-regulation of α-SMA, collagen IV, and fibronectin in UUO mouse model of kidney fibrosis, suggesting that SIRT1 activity may be essential in preventing TGF-β1 induced fibrotic response via Smad3 deacetylation (50).

### Renin-angiotensin system and SIRT1

Renin–angiotensin system, especially Angiotensin II (AngII) is closely associated with the development and progression of DKD. RAS inhibition by angiotensin-converting enzyme inhibitors (ACEi), which inhibits the conversion of AngI to AngII, or angiotensin receptors blockers (ARBs) decreases proteinuria in patients with diabetic nephropathy and halts the disease progression to ESRD, thus improving patient survival (82). Angiotensin-converting enzyme 2 (ACE2), a homolog of ACE not inhibited by ACEi, counteracts AngII effects by hydrolyzing AngII into Angiotensin 1–7 (Ang 1–7), which in turn protects against DKD by increasing tissue triglyceride degradation and decreasing kidney weight, mesangial expansion, proteinuria, and renal fibrosis (4). SIRT1 regulates RAS by binding and activating ACE2 promoter, leading to increased Ang 1–7 concentrations (4, 44). It also promotes vasodilatation by decreasing the expression of the potent vasoconstrictor angiotensin II receptor-type I (AT_1_R), and protects vascular tissues through increased nitric oxide production by deacetylating eNOS in endothelial cells (58).

### Water handling

Diabetes mellitus is associated with a significant polyuria and natriuresis, as well as increased plasma aldosterone and anti-diuretic hormone arginine vasopressin (AVP) levels. Studies have identified serum and glucocorticoid induced kinase-1 (SGK-1) as a key signaling element in diabetic nephropathy (83, 84). Under the hyperglycemic state the increase in Ca^2+^ and TGF-β1 leads to upregulation of SGK-1 in kidney, which regulates epithelial Na^+^ channel (ENaC) activation, leading to increased sodium absorption (85). A physiological role for SIRT1 in regulating the α-ENaC expression has been reported in cultured renal inner medullary collecting duct cells (mIMCD3) (55). SIRT1 interacts with Dot (disruptor of telomeric silencing)-1, a histone H3K79 methyltransferase, and enhances its activities on histone H3K79 methylation in chromatin along the α-ENaC promoter, and thereby repressing α-ENaC transcription in mIMCD3 cells (55).

## Conclusion and Prospects

Since the first description of the Sir2 family and its effects on longevity in yeast, our understanding of the specific actions and role of SIRT1 on different kidney diseases have increased tremendously. Administration of SIRT1 activators showed a restoration of SIRT1 levels, decreased albuminuria, glomerular hypertrophy, and kidney fibrosis in DKD models (86–90). The mechanisms by which these activators exert their beneficiary effects are currently under extensive research, highlighting the importance of developing therapies to increase either SIRT1 expression or activity in patients with DKD to prevent disease progression. In addition, single nucleotide polymorphisms (SNPs) within the gene encoding SIRT1 have shown to have a directionally consistent association with diabetic nephropathy leading to the assumption that SIRT1 not only play a protective role, but certain SNPs variations of SIRT1 might predispose an individual to DKD (91). These findings warrant further investigations into the functions of these SNPs variations, and to develop new strategies for protection against renal diseases.

## Conflict of Interest Statement

The authors declare that the research was conducted in the absence of any commercial or financial relationships that could be construed as a potential conflict of interest.
